# A compact VEGF signature associated with distant metastases and poor outcomes

**DOI:** 10.1186/1741-7015-7-9

**Published:** 2009-03-16

**Authors:** Zhiyuan Hu, Cheng Fan, Chad Livasy, Xiaping He, Daniel S Oh, Matthew G Ewend, Lisa A Carey, Subbaya Subramanian, Robert West, Francis Ikpatt, Olufunmilayo I Olopade, Matt van de Rijn, Charles M Perou

**Affiliations:** 1 Lineberger Comprehensive Cancer Center, University of North Carolina at Chapel Hill, Chapel Hill, NC 27599, USA; 2 Department of Genetics, University of North Carolina at Chapel Hill, Chapel Hill, NC 27599, USA; 3 Department of Pathology and Laboratory Medicine, University of North Carolina at Chapel Hill, Chapel Hill, NC 27599, USA; 4 Department of Medicine, University of North Carolina at Chapel Hill, Chapel Hill, NC 27599, USA; 5 Department of Pathology, Stanford University Medical Center, Stanford, CA 94035, USA; 6 Section of Hematology/Oncology, Department of Medicine, Committees on Genetics and Cancer Biology, University of Chicago, South Maryland Avenue, Chicago, IL 60637-1463, USA

## Abstract

**Background:**

Tumor metastases pose the greatest threat to a patient's survival, and thus, understanding the biology of disseminated cancer cells is critical for developing effective therapies.

**Methods:**

Microarrays and immunohistochemistry were used to analyze primary breast tumors, regional (lymph node) metastases, and distant metastases in order to identify biological features associated with distant metastases.

**Results:**

When compared with each other, primary tumors and regional metastases showed statistically indistinguishable gene expression patterns. Supervised analyses comparing patients with distant metastases versus primary tumors or regional metastases showed that the distant metastases were distinct and distinguished by the lack of expression of fibroblast/mesenchymal genes, and by the high expression of a 13-gene profile (that is, the 'vascular endothelial growth factor (VEGF) profile') that included *VEGF, ANGPTL4, ADM *and the monocarboxylic acid transporter *SLC16A3*. At least 8 out of 13 of these genes contained HIF1α binding sites, many are known to be HIF1α-regulated, and expression of the VEGF profile correlated with HIF1α IHC positivity. The VEGF profile also showed prognostic significance on tests of sets of patients with breast and lung cancer and glioblastomas, and was an independent predictor of outcomes in primary breast cancers when tested in models that contained other prognostic gene expression profiles and clinical variables.

**Conclusion:**

These data identify a compact *in vivo *hypoxia signature that tends to be present in distant metastasis samples, and which portends a poor outcome in multiple tumor types.

This signature suggests that the response to hypoxia includes the ability to promote new blood and lymphatic vessel formation, and that the dual targeting of multiple cell types and pathways will be needed to prevent metastatic spread.

## Background

Metastases are the main cause of mortality for patients with breast cancer. The molecular biology behind metastasis is complex and likely requires changes in cell cycle regulation [1], the repertoire of expressed proteases and protease inhibitors [2], proteins that promote autocrine growth loops, and/or proteins that cause an epithelial-to-mesenchymal transition [3]. To make matters more complicated, it is clear that metastasis biology is in part governed by non-tumor cells including fibroblasts [4], endothelial cells [5], and myoepithelial cells [6]. For example, recent evidence suggests that tumor endothelial cell interactions are important for determining patient outcomes as evidenced by the promising results from clinical trials that use bevacizumab, a monoclonal antibody directed against vascular endothelial growth factor (VEGF) [7,8].

Genomic profiling of human tumors and model systems has identified important features concerning metastasis biology. First, it has been shown that the expression profile of primary tumors without metastases can be highly predictive of the development of future metastases [9-13]. Second, cell lines can be selected that have specific end-organ tropisms with distinct expression profiles [14,15]. Finally, cell line and murine models have demonstrated many different genes as being important for breast tumor metastasis, including *Twist *[16], *Snail *[3], and *CXCL12 *[17]. In this paper, we compare primary breast tumors, regional metastases, and distant metastases with each other and show that distant metastasis samples are distinct and provide unique signatures that predict poor outcomes in primary tumors.

## Methods

### Tissue samples and microarray protocols

One hundred and forty-six patients represented by 161 breast tumor specimens (with 23 paired tumor samples) and 10 normal breast samples (195 total microarrays) were profiled. Most of these samples appeared in previous publications [18-20], with 39 being new to this study, and all of which were collected using institutional review board-approved protocols. The clinical information for all samples is in the table in Additional file 1. Included within the 161 profiled tumors were 134 primary tumors, nine regional metastases and 18 distant metastases. Patients were heterogeneously treated in accordance with the standard of care dictated by their disease stage, estrogen receptor (ER) and HER2 status.

Total RNA isolation and microarray protocols are described in Hu et al [21]. Each sample was assayed versus a common reference sample [22]. The microarrays used were Agilent Human oligonucleotide microarrays that were scanned on an Axon GenePix 4000B, analyzed with GenePix Pro 4.1, and Lowess normalized. All microarray data have been deposited into the GEO under the accession number of GSE3521.

### Supervised microarray data analysis

The background-subtracted, Lowess-normalized log_2 _ratio of Cy5 over Cy3 intensity values were filtered to select genes that had a signal intensity of > 30 units in both the Cy5 and Cy3 channels. Only genes that met these criteria in at least 70% of the 195 microarrays were included for subsequent analysis. Next, each patient was classified according to the following metastasis scoring system (MetScore): MetScore = 1 were patients that had a primary tumor and were clinically node negative (*N *= 0) and distant metastasis negative (*M *= 0); MetScore = 2 were patients that had a regional metastasis (*N *= 1–3) and no distant metastasis (*M *= 0); MetScore = 3 were patients with confirmed distant disease at the time of diagnosis (*M *= 1 and any *N*) or that were represented by an actual distant metastasis sample. We next performed a multi-class significance analysis of microarrays (SAM) using a single sample from each patient, biasing the sample selection to use the actual regional or distant metastasis samples (146 arrays, see Additional file 1). We identified the gene set that was associated with the MetScore 1-2-3 distinction, which gave 1195 genes at a false discovery rate of 5%. This gene set was next used in a one-way average linkage hierarchical cluster using the program 'Cluster' [23], with the data being displayed relative to the median expression for each gene using 'Java Treeview' [24].

### Cross-validation analyses

Relationships between the gene expression data and the MetScore classification was further examined using a 10-fold cross-validation (CV) analysis to identify a set of genes that might distinguish a MetScore group from the others. 10-fold CV using five different statistical predictors including PAM [25], a *k*-nearest neighbor classifier with either Euclidean distance or one-minus-Spearman-correlation as the distance function, and a class nearest centroid metric with either Euclidean distance or one-minus-Spearman-correlation as the distance function, were used as described in Chung et al [26]. We performed 10-fold CV using the five different statistical predictors with the reported CV prediction accuracies being the average of the five predictors (Tables 1, 2, 3 and 4).

**Table 1:** Cox proportional hazards models for relapse-free survival using the NKI 295 patient test data set – model containing the clinical variables and the VEGF profile

**Variable**	**DF**	**Estimate**	**Standard Error**	**Chi-Square**	**Pr > ChiSq**	**Hazard Ratio**	**95% Hazard Ratio Confidence Limits**	
Age	1	-0.05508	0.01622	11.5365	0.0007	0.946	0.917	0.977
ER	1	-0.12785	0.23563	0.2944	0.5874	0.88	0.555	1.397
Grade2vs1	1	0.8058	0.31181	6.6784	0.0098	2.238	1.215	4.124
Grade3vs1	1	0.76706	0.32265	5.6519	0.0174	2.153	1.144	4.053
Tsize	1	0.37409	0.19444	3.7017	0.0544	1.454	0.993	2.128
node	1	0.33066	0.17801	3.4504	0.0632	1.392	0.982	1.973
Treatment	1	-0.65688	0.27811	5.5788	0.0182	0.518	0.301	0.894
**VEGF_3group**	**1**	**0.47238**	**0.14838**	**10.1355**	**0.0015**	**1.604**	**1.199**	**2.145**

**Table 2:** Cox proportional hazards models for relapse-free survival using the NKI 295 patient test data set – model containing the clinical variables and multiple gene expression profiles

**Variable**	**DF**	**Estimate**	**Standard Error**	**Chi-Square**	**Pr > ChiSq**	**Hazard Ratio**	**95% Hazard Ratio Confidence Limits**	
Age	1	-0.0505	0.0174	8.4217	0.0037	0.951	0.919	0.984
ER	1	-0.5654	0.34723	2.6514	0.1035	0.568	0.288	1.122
Grade2vs1	1	0.15563	0.33471	0.2162	0.642	1.168	0.606	2.252
Grade3vs1	1	0.02327	0.36156	0.0041	0.9487	1.024	0.504	2.079
Tsize	1	0.53014	0.19935	7.0723	0.0078	1.699	1.15	2.511
node	1	0.15863	0.19203	0.6824	0.4087	1.172	0.804	1.707
Treatment	1	-0.63284	0.29747	4.526	0.0334	0.531	0.296	0.951
**VEGF_3group**	**1**	**0.47637**	**0.1597**	**8.8972**	**0.0029**	**1.61**	**1.177**	**2.202**
GHI	1	0.24924	0.22057	1.2769	0.2585	1.283	0.833	1.977
Gene70	1	0.6283	0.33298	3.5605	0.0592	1.874	0.976	3.6
Wound_Response	1	0.8087	0.35969	5.0549	0.0246	2.245	1.109	4.543
LumA_LumB	1	0.74421	0.36168	4.2339	0.0396	2.105	1.036	4.276
LumA_Basal	1	-0.68615	0.48782	1.9785	0.1596	0.504	0.194	1.31
LumA_Her2- enrich	1	-0.16349	0.45232	0.1306	0.7178	0.849	0.35	2.061
LumA_Normal	1	0.51006	0.37635	1.8368	0.1753	1.665	0.796	3.482
ER-regulated	1	0.02629	0.32219	0.0067	0.935	1.027	0.546	1.93
TP53-associated	1	-0.0764	0.31658	0.0582	0.8093	0.926	0.498	1.723

**Table 3:** Cox proportional hazards models for relapse-free survival using the NKI 295 patient test data set – backwards selected model from Table 2B showing the final parameters

**Variable**	**DF**	**Estimate**	**Standard Error**	**Chi-Square**	**Pr > ChiSq**	**Hazard Ratio**	**95% Hazard Ratio Confidence Limits**	
Age	1	-0.05279 0.01751		9.0867	0.0026	0.949	0.917	0.982
Tsize	1	0.50134	0.19171	6.8387	0.0089	1.651	1.134	2.404
Treatment	1	-0.48902 0.19731		6.1425	0.0132	0.613	0.417	0.903
**VEGF_3group**	**1**	**0.46592**	**0.14522**	**10.2939**	**0.0013**	**1.593**	**1.199**	**2.118**
Gene70	1	0.80232	0.27062	8.7898	0.003	2.231	1.312	3.791
Wound_Response	1	0.89085	0.33694	6.9903	0.0082	2.437	1.259	4.717
LumA_LumB	1	0.8682	0.23035	14.2059	0.0002	2.383	1.517	3.742
LumA_Normal	1	0.67587	0.29743	5.1638	0.0231	1.966	1.097	3.521

**Table 4:** Cox proportional hazards models for relapse-free survival using the NKI 295 patient test data set – model containing the clinical variables and the VEGF-profile as a continuous variable

**Variable**	**DF**	**Estimate**	**Standard Error**	**Chi-Square**	**Pr > ChiSq**	**Hazard Ratio**	**95% Hazard Ratio Confidence Limits**	
Age	1	-0.0589	0.01639	12.906	0.0003	0.943	0.913	0.974
ER	1	0.00607	0.25196	0.0006	0.9808	1.006	0.614	1.649
Grade2vs1	1	0.82592	0.31135	7.0367	0.008	2.284	1.241	4.204
Grade3vs1	1	0.78226	0.32128	5.9284	0.0149	2.186	1.165	4.104
Tsize	1	0.31513	0.19566	2.5941	0.1073	1.37	0.934	2.011
node	1	0.3314	0.17655	3.5233	0.0605	1.393	0.985	1.969
Treatment	1	-0.61996	0.27417	5.1133	0.0237	0.538	0.314	0.921
**VEGF_Continuous**	**1**	**0.43301**	**0.12298**	**12.3972**	**0.0004**	**1.542**	**1.212**	**1.962**

### VEGF profile analyses

For the VEGF profile, an average expression value across all 13 genes (*RRAGD, FABP5, UCHL1, GAL, PLOD, DDIT4, VEGF, ADM, ANGPTL4, NDRG1, NP, SLC16A3 *and *C14ORF58*) was determined and the patients were placed into a three-group classification based their 13-gene average log_2 _expression ratio from the University of North Carolina (UNC) training data set and using the cut off values (-0.63/0.08) that were identified using X-tile [27] and relapse-free survival as the endpoint. Analyses using the VEGF profile and the training set cutoffs were also applied to an independent test set of 295 patients assayed on Agilent microarrays (that is, NKI295 [28]), to a set of lung carcinoma samples from Bhattacharjee et al [29], and to the glioblastoma sample set from Nutt et al [30]. To perform these across-data set analyses, for the NKI295 dataset we used the log ratio of red channel intensity versus green channel intensity and the data was median centered for every gene across the 295 arrays. The Netherlands Cancer Institute (NKI) dataset was then distant weight discrimination (DWD) normalized [31] with the UNC training dataset after collapsing by NCBI Entrez GeneID; after DWD normalization, the NKI data was also column standardized. For the Affymetrix datasets the probe level intensity .CEL files were processed by robust multi-chip average. The probe sets' log intensity was median centered for every gene across all the arrays. The Affymetrix datasets were also DWD normalized relative to the UNC training data after collapsing by NCBI Entrez GeneID, and were column standardized.

#### ***Multiple expression predictor analyses***

First, each sample was assigned an 'intrinsic subtype' as described in Hu et al [18], where a centroid was created for each of the following intrinsic subtypes: Basal-like, Luminal A, Luminal B, HER2-enriched and Normal-like. Next, we tested for associations between a tumor's intrinsic subtype, the VEGF profile and other published expression profiles implicated in metastasis biology that included a) the 70-gene outcome predictor developed by van't Veer et al [10,11], b) the 'wound-response' profile [32], c) the hypoxia-induced cell line signature [33], d) the 11-gene BMI/stem cell signature [34], e) a bone metastasis signature [14], f) a lung metastasis signature [15], and g) the expression profiles of *HIF1α*, *Snail *[3] and *Twist *[16]; we extracted as many genes as was possible from our microarrays for each predictor and followed the classification scheme described by the authors. For the bone metastasis signature [14], we created an average value for each patient using the 43 genes that were highly expressed in the cell line derivatives that metastasized to the bone; we performed a similar analysis for the lung metastasis signature [15].

Lastly, for the 11-gene stem cell signature [34], we created an average value across all 11 genes. We also created a 'glycolysis-profile' by starting with the nine glycolysis genes/probes present on the array, then filtering for probes that showed > 30 intensity units in both channels, and then selecting for 70% good data across all samples; next we selected the subset of glycolysis gene probes that passed filtering and showed a Pearson correlation of greater than 0.4, which resulted in the selection of six out of nine glycolysis genes (*GPI, PKM2, PFKP, PGK1, GAPD, ENO1*), which were then used to create an average profile for each patient.

We examined correlations between profiles using multiple methods (Additional file 1): for quantized profile testing, Chi-squared analysis and Fischer's exact test were used. For continuous variable testing, ANOVA analyses were performed. Finally, we also performed a calculation of the Cramer's V statistic for the evaluation of the strength of association between two quantized variables (see Oh et al [19]).

### Survival analyses

Univariate Kaplan-Meier analysis was performed with a log-rank test using WinSTAT for excel. Multivariate analysis of the NKI295 test set using Cox proportional hazards modeling was conducted in SAS version 9.1; a Cox hazard model was tested that included estrogen receptor status (coded as positive vs. negative), tumor size (coded as ≤ 2 cm vs. > 2 cm), lymph node status (codes as 0, 1–3, > 3 positive nodes or *M *= 1), age (continuous variable, formatted in decades), grade (coded as grade 1 vs. 2, and grade 1 vs. 3), and treatment (coded as yes if treatment with chemo and/or hormonal therapy, no if no adjuvant therapy was given), and the VEGF profile of low, intermediate or high as a single categorical variable. Another Cox model was also tested that included all the clinical variables, the VEGF profile, and other expression predictors [11,13,18,19,28,35].

### In situ hybridization and immunohistochemistry

*In situ *hybridization (ISH) on tissue microarrays containing 250 different human breast tumors (not related to the 146 patients used for microarray analysis) was performed as previously described [36]. Briefly, digoxigenin (DIG)-labeled sense and anti-sense RNA probes are generated by PCR amplification of approximately 450 bp products with the T7 promoter incorporated into the primers; the primer sequences used for amplification were VEGF (Forward-tctccctgatcggtgacagt, Reverse-tcgaaaaactgcactagagacaa), ANGPTL4 (Forward: gggaatcttctggaagacctg, Reverse-tacacacaacagcaccagca) and ADM (Forward-gtgtttgccaggcttaagga, Reverse-tcggtgtttccttcttccac). *In vitro *transcription was performed with a DIG RNA-labeling kit and T7 polymerase according to the manufacturer's protocol (Roche Diagnostics, Indianapolis, IN). Immunohistochemistry (IHC) was performed for HIF1α using Mouse Anti-Human HIF1α (BD Biosciences #610958) according to the protocol from Vleugel et al [37]; the tumors were scored for perinecrotic and diffuse staining as described in Vleugel et al.

## Results

### Expression patterns associated with metastases

**Figure 1:**
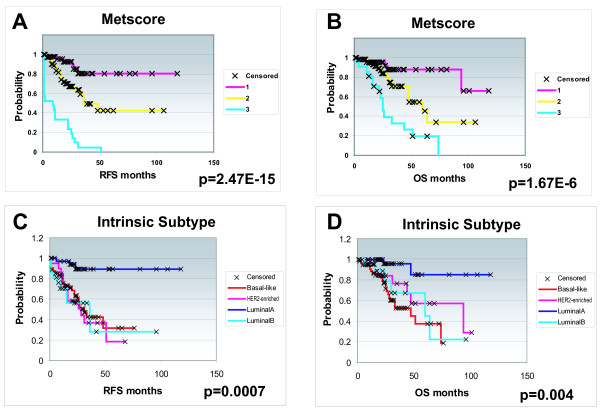
**Kaplan-Meier survival plots**. Kaplan-Meier survival plots according to MetScore status (**A and B**) and according to intrinsic subtype (**C and D**) across the 146 patient UNC training data set.

To identify gene expression patterns associated with breast cancer metastases, we performed 195 microarrays representing 134 primary tumors, nine regional metastases and 18 distant metastasis specimens (146 different patients and 10 normal breast tissues). Each patient was classified according to a MetScore, which is roughly analogous to stage except that tumor size was not considered (see Methods). As expected, this scoring system was highly predictive of patient outcomes (Figure 1A and 1B). Using the MetScore classifications, we performed CV analyses to determine if any MetScore group might be distinct relative to the others. Low accuracy rates (56% to 65%) for the prediction of MetScore 1 vs. MetScore 2 specimens were observed; however, when MetScore 1 vs. MetScore 3 (80% to 85%) or MetScore 2 vs. MetScore 3 samples (81% to 83%) were compared, high accuracy rates were obtained, which suggests that MetScore 3 was the most distinct group.

**Figure 2:**
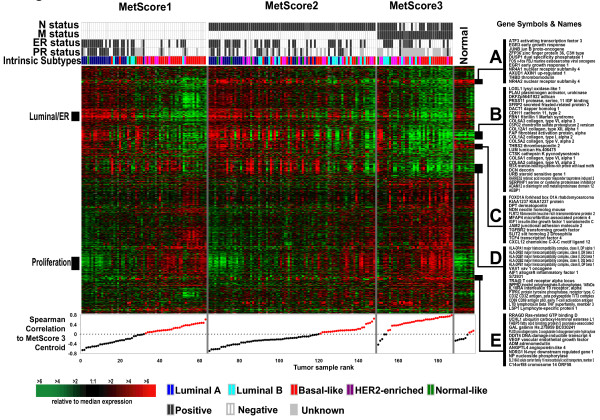
**One-way average linkage hierarchical cluster analysis**. One-way average linkage hierarchical cluster analysis of the gene set associated with MetScore status. One hundred and ninety-five microarrays, representing 146 tumors and 10 normal breast samples were analyzed using the 1195 gene MetScore gene set. Overview of the complete cluster diagram (the full cluster diagram can be found as Additional file 2). The tumors were ordered according to their MetScore, and then according to their increasing correlation to the Metscore3 centroid within each group. Clinical regional node status, distant metastasis status, ER, PR, and intrinsic subtype are shown. **A) **FOS-JUN gene expression cluster, **B) **fibroblast/mesenchymal cell cluster, **C) **CXCL12 gene expression cluster, **D)**, immune-cell/HLA cluster, **E) **VEGF profile.

Next, we performed a multi-class significance analysis of microarray [38] analysis using a single sample from each of the 146 patients and the MetScore 1-2-3 grouping and obtained a 1195 genes at a 5% false discovery rate. This gene set was then used in a one-way average linkage hierarchical clustering analysis (Figure 2 and Additional file 2) where the samples were first ordered according to MetScore, and then according to their correlation to the average profile (that is, centroid) of the MetScore 3 class. This analysis demonstrates that some MetScore 1 and 2 samples actually have a MetScore 3 profile; a similar result has been shown before by Ramaswamy et al [9].

The gene expression patterns from this SAM analysis were complex and there were few, if any, that directly correlated with a simple progression from MetScore 1 to 2 to 3. Included within this gene set were many clusters and/or gene sets that have been identified previously, including a luminal/ER+ pattern [11,39,40] and a proliferation signature [41,42], both of which are integral parts of a gene expression assay that predicts the likelihood of recurrence in ER+ and patients treated with tamoxifen [13]. In addition, many other biologically important gene sets were identified, including an 'immediate early' gene cluster containing *c-FOS *and *JUNB *(Figure 2A) [43], a set of fibroblast genes containing *PLAU*, *THSB2 *and multiple collagen genes (Figure 2B), a set of immune cell genes (Figure 2D), and a gene set containing *CXCL12 *(Figure 2C); *CXCL12 *was the top-ranked gene from this SAM analysis and was recently identified as a chemokine whose high expression promotes tumor cell proliferation, migration and invasion [17]. Analysis of these individual clusters by EASE [44], with both EASE score and Bonferroni < 0.05 used as the cut off, identified many significant gene ontology categories that included 'transcription regulation' and 'DNA/nucleic acid binding' for the *FOS-JUN *cluster, while the fibroblast cluster was over-represented for 'extracellular matrix', 'cell adhesion and communication', 'organogenesis', 'development', and 'regulation of protease activity'. The *CXCL12 *cluster was over-represented for 'cell adhesion', 'cell migration' and 'extracellular matrix'. Lastly, a small 13-gene cluster containing *VEGF*, *Adrenomedulin *(*ADM*) and *Angiopoietin-like 4 *(*ANGPTL4*) was identified as the 'VEGF-profile' (Figure 2E), which is discussed below in greater detail.

Our previous work identified five 'intrinsic' subtypes of breast cancer that are of prognostic and predictive value [18,41,45]. Subtype classification of the tumors using the centroid predictor from Hu et al [18] showed significant outcome predictions (Figure 1C and 1D). A Chi-squared test (*p *= 0.0006) showed that intrinsic subtype was significantly correlated with MetScore, with the Basal-like and HER2-enriched groups being the most frequent in MetScore 3 and with no Luminal A samples being in the MetScore 3 group. Correlations between tumor subtype and stage have been described [46,47], and were recapitulated here.

### Analysis of the VEGF profile

**Figure 3:**
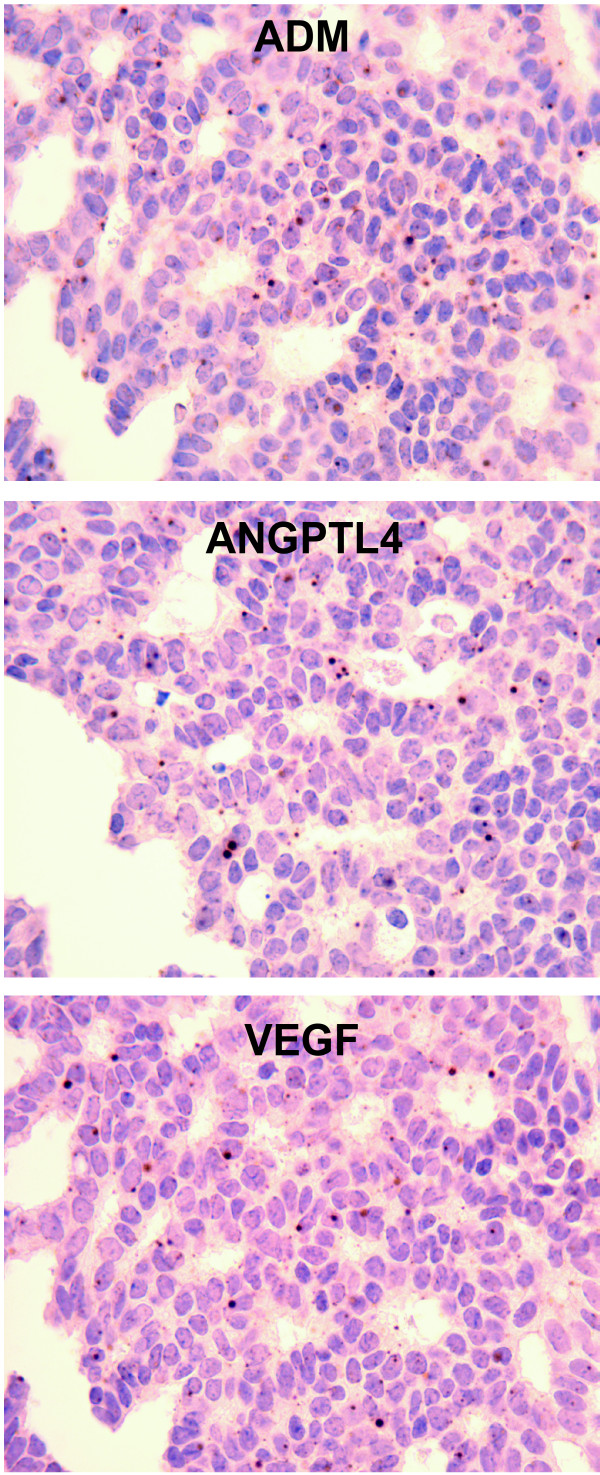
***In situ *hybridization**. *In Situ *hybridization to localize gene transcripts using a representative tumor for **A) **Adrenomedulin (ADM), **B) **Angiopoetin-like 4 (ANGPLT4), and **C) **Vascular Endothelial Growth Factor A (VEGF). Magnification 200×.

A small cluster of genes containing *VEGF *was identified (Figure 2E) that showed high expression in MetScore 3 tumors. This gene cluster contained several secreted proteins that have been implicated in endothelial cell (VEGF and ANGPTL4), lymphatic cell (ADM) and smooth muscle cell (GAL) dynamics. As a step in evaluating this profile, we performed ISH to determine what cell type was producing VEGF, ANGPTL4 and ADM. In the vast majority of cases that showed strong ISH positivity (which totaled approximately 10% of the 250 tumors tested), it was the tumor cells themselves that produced the mRNA for these three genes, and typically all three were produced (Figure 3). In a few cases, both tumor and fibroblasts showed ISH positivity, but this was rare.

**Figure 4:**
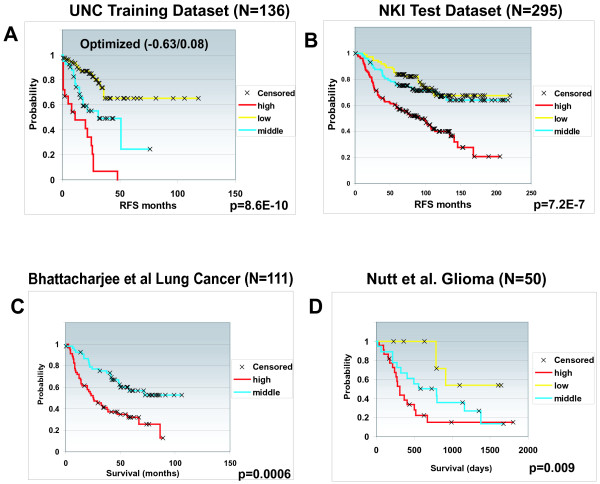
**Univariate Kaplan-Meier survival plots**. Univariate Kaplan-Meier survival plots of survival for patients stratified using the VEGF profile on the **A) **UNC training data set, **B) **NKI test data set, **C) **Bhattacharjee et al lung carcinoma data set [29], and **D) **Nutt et al glioblastoma data set [30]. Note: two genes *ANGPTL4 *and *C14ORF58 *were not found on Affymetrix platforms for C and D.

As a second step in the evaluation of the VEGF profile, we created an average expression ratio of the 13 genes for each patient and looked for correlations with outcome. By dividing the patients into low, intermediate and high-expression groups using relapse-free survival (RFS) and cutoffs determined by X-tile [27], we saw that the VEGF profile was prognostic of RFS (Figure 4A) and overall survival (data not shown) with the high expression portending a poor outcome. Rank order expression classifications (two or three groups) were also robust methods of predicting outcomes (Additional file 3). Applying the VEGF profile classification rules to an independent test set of 295 patients (*that is*, NKI295) [10,28] also significantly predicted outcomes (Figure 4B), as did rank order classifications (Additional file 3). This classification rule was also of prognostic value on a set of lung carcinoma samples (Figure 4C and Additional file 3E and 3F), although there were too few 'low' samples to be included into the Kaplan-Meier plot analysis, and on a set of patients with glioblastoma (Figure 4D and Additional file 3G and 3H); we noted that two genes (*ANGPTL4 *and *C14ORF58*) were not found on the Affymetrix platform for lung and glioblastoma test data sets. However, the Pearson correlation is 0.992 (UNC training dataset) and 0.986 (NKI295 test dataset) respectively between the average of the 13 genes and that of the 11 genes (omitting *ANGPTL4 *and *C14ORF58*). We repeated the survival analysis for the UNC dataset and NKI test set again using the 11 genes and the results were very similar (data not shown).

A multivariate Cox proportional hazards analysis on the NKI295 test set using RFS was performed using clinical variables and the VEGF profile, and it was determined that the VEGF profile was a significant predictor of RFS (Table 1). In Fan et al [48], we evaluated the prognostic powers and concordance across multiple expression predictors including the intrinsic subtypes, the NKI 70 gene signature, a microarray-based version of the Genomic Health Inc. Recurrence Score, and the wound-response profile using this same NKI patient data set, and we have also identified other profiles of prognostic significance including an estrogen pathway [19] and p53 mutation profiles [35]; therefore, we performed a Cox proportional hazards analysis (Table 2) with backwards variable selection (Table 3) to evaluate a model that contained all of the aforementioned gene expression predictors and clinical variables. The final model contained both clinical parameters and multiple gene expression predictors including the VEGF profile (Table 3). Similar results were obtained when using time to distant metastasis formation, or overall survival (data not shown), or when treating the VEGF profile as a continuous variable (Table 4).

### Analysis of a glycolysis-profile and HIF1α expression

**Figure 5:**
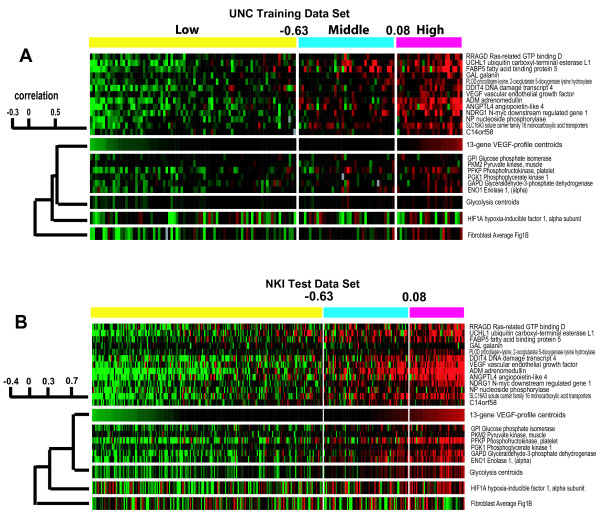
**VEGF profile, glycolysis and HIF1α gene expression analyses**. **A) **Gene expression for the VEGF profile (plus average values), for the six glycolysis genes and glycolysis centroid, HIF1α and fibroblast centroids are shown across the 146 patient UNC training data set with the tumors ordered according to their VEGF profile average values. **B) **Similar analysis as presented in A except the data set is the NKI patient test set.

A biological implication of the VEGF profile is that it is related to a tumor's response to hypoxic conditions, which historically has been referred to as the Warburg effect [49,50]. A central tenant of the Warburg effect is that a tumor's metabolism becomes more dependent upon glycolysis due to anaerobic conditions. To examine glycolysis using a genomic approach, we created a 'glycolysis-profile' using the six most highly correlated glycolytic enzyme probes (GPI, PKM2, PFKP, PGK1, GAPD, ENO1, Figure 5A); the VEGF profile and the six best glycolysis probes were highly correlated (*p *< 0.001, Table 5).

**Table 5:** Correlation analysis of multiple gene expression profiles linked to metastasis biology or formation compared with each other

**Quantized Variables Testing**				
**Primary Signature**	**Test Signature**	**Ch-aquare *P*-value**	**Cramer's V**	**Fisher Exact *P*-value**

VEGF profile	MetScore	0.0002	0.272	4.80E-04
VEGF profile	NKI 70-gene profile	0.0008	0.3126	3.60E-04
VEGF profile	Wound Response Profile	0.0001	0.3524	3.78E-06
VEGF profile	Intrinsic Subtype	< 0.0001	0.4223	4.29E-11
VEGF profile	hypoxia-signature	< 0.0001	0.6394	1.10E-15
VEGF profile	hypoxia-metagene (50:50)	< 0.0001	0.5722	8.29E-12
				
Intrinsic Subtype	MetScore	0.0054	0.2578	7.09E-04
Intrinsic Subtype	Hypoxia signature	< 0.0001	0.739	1.40E-20
Intrinsic Subtype	VEGF profile	< 0.0001	0.4223	4.29E-11
Intrinsic Subtype	NKI 70-gene profile	< 0.0001	0.4449	5.94E-06
Intrinsic Subtype	Wound Response Profile	< 0.0001	0.7389	1.56E-16
Intrinsic Subtype	Hypoxia metagene (50:50)	< 0.0001	0.5181	4.91E-09

**Continuous Variables Testing**				

**Primary Signature**	**Test Signature**	**ANOVA *P*-value**		

VEGF profile	BoneMeta 43 Up genes Average	< 0.0001		
VEGF profile	Breast2Lung-Average	< 0.0001		
VEGF profile	Snail1	< 0.0001		
VEGF profile	Twist1	0.3		
VEGF profile	11 stem cell signature-Average	0.0074		
VEGF profile	6-Best-Glycolysis-Probes	< 0.0001		
VEGF profile	Fibroblast-line-Avg	0.7		
VEGF profile	HIF1A	0.0004		
VEGF profile	hypoxia-metagene (50:50)	< 0.0001		
				
Intrinsic Subtype	BoneMeta 43 Up genes Average	0.054		
Intrinsic Subtype	Breast2Lung-Average	0.036		
Intrinsic Subtype	Snail1	0.0002		
Intrinsic Subtype	Twist1	0.2		
Intrinsic Subtype	11 stem cell signature-Average	< 0.0001		
Intrinsic Subtype	6-Best-Glycolysis-Probes	< 0.0001		
Intrinsic Subtype	Fibroblast-line-Avg	0.012		
Intrinsic Subtype	HIF1A	0.0033		
Intrinsic Subtype	hypoxia-metagene (50:50)	< 0.0001		

HIF1α is a known regulator of VEGF expression, and therefore we determined that HIF1α mRNA gene expression was correlated with the VEGF profile (*p *= 0.0004; Table 5); in addition, 'perinecrotic' HIF1α IHC staining as defined by Vleugel et al [37] was also assayed on a subset of 66 of these tumors and was correlated with expression of the VEGF profile (ANOVA *p*-value = 0.018, data not shown), while a 'diffuse' HIF1α IHC profile was not. Next, the promoter region of each of the genes in the VEGF profile was examined using the program rVISTA [51] and showed that *DDIT4, VEGF, NDRG1, SLC16A3, PLOD, ADM, ANGPTL4 *and *C14ORF58 *all had hypoxia response elements within 2000 bp upstream of their start codons; it is already known that many of these genes including *VEGF *[52], *ADM *[53], and *DDIT4 *[54] are HIF1α-regulated. Nearly identical genomic results were also obtained from the NKI295 test set (Figure 5B).

### Fibroblast signature

**Figure 6:**
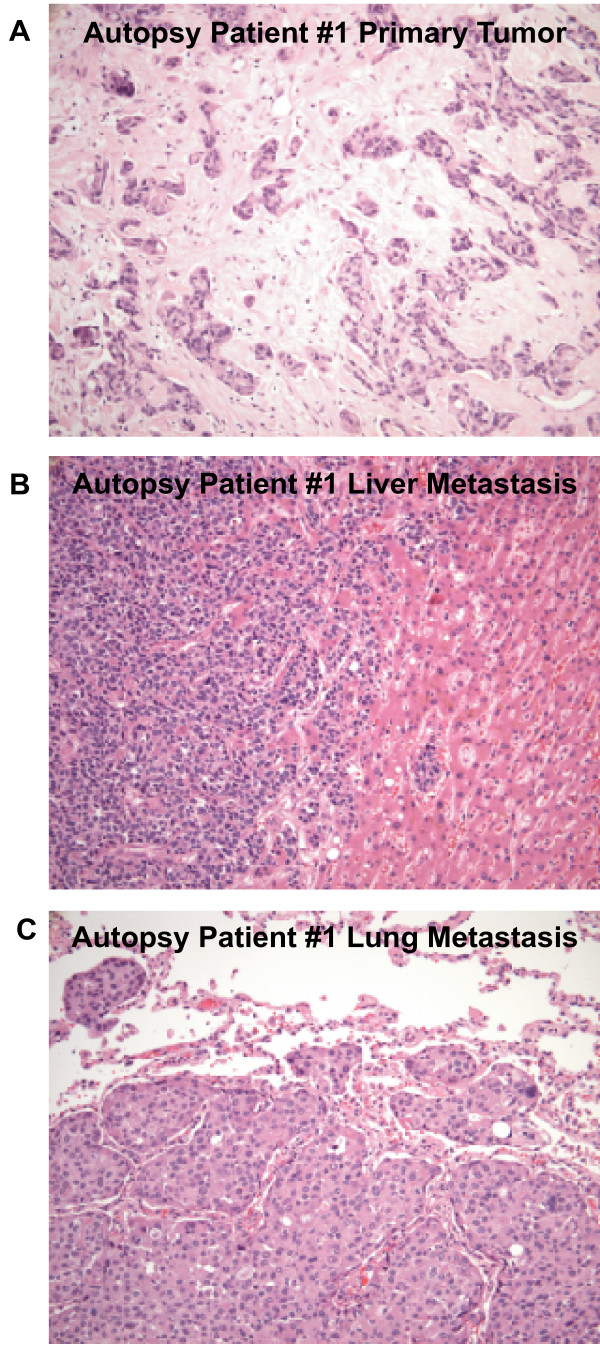
**H&E images of a primary breast tumor taken from a MetScore 3 patient**. Showing a prominent admixed stromal component comprised of fibroblasts and myofibroblasts in the primary tumor. The fibroblast/myofibroblast component is markedly diminished in the distant metastatic sites (**B and C**) as compared with the primary tumor (**A**). Magnification 200×.

A fibroblast/mesenchymal signature was another profile that changed with MetScore (Figure 2B), and thus to examine the potential fibroblast cell content present within each MetScore group we determined each patient's average expression value of the genes contained with the cluster presented in Figure 2B. This gene set contains fibrillin, fibroblast activation protein alpha, six collagen protein subunits and versican, which are genes and/or proteins that are typically produced by fibroblast and/or mesenchymal cells [55]. This analysis shows that the fibroblast profile is correlated with intrinsic subtype (Table 5, *p *= 0.012) and that the MetScore 3 samples had the lowest average expression compared with the MetScore 1 and 2 samples (ANOVA *p*-value = 0.005, data not shown). Pathological examination of H&E sections of the distant metastasis samples also supports this conclusion and shows scant admixed mesenchymal cells in the distant metastasis samples versus their primaries that show abundant admixed mesenchymal cells (Figure 6).

### Correlations between multiple metastasis associated profiles

We examined whether the intrinsic subtypes, the MetScore classification, and the VEGF signature correlated with any of the following expression profiles that have been associated with metastatic potential: a) the NKI 70-gene predictor [10,11], b) the 'wound-response' profile [32], c) a cell line-derived hypoxia profile [33], d) an 11-gene BMI/stem cell signature [34], e) a bone metastasis signature [14], f) a lung metastasis signature [15], g) a hypoxia metagene [56], and h) the expression profile of three individual genes (*HIF1α*, *Snail *[3], and *Twist *[16]). These analyses identified a large amount of concordance across profiles (Table 5). For example, the breast tumor subtype was significantly correlated with the bone and lung profiles, *Snail *expression, and the 11-gene stem cell signature; in particular, the bone and lung profiles were associated with both ER-negative subtypes (Basal-like and HER2-enriched), and *Snail *expression and the 11-gene stem cell signature were the highest within the Basal-like subtype. Similar results were also observed when the VEGF profile was compared with the other profiles. Two 'hypoxia signatures' have been described and shown to be of prognostic value across a variety of tumor types including breast [33,56]; the large signature of Chi et al [38] showed a four-gene overlap with the VEGF profile (*ADM, NDRG1, DDIT4 *and *ANGPLT4*) while the 'hypoxia-metagene' of Winter et al [56] showed a three-gene overlap (*VEGF, NDRG1 *and *ANGPLT4*); as might be expected, all three of these profiles were correlated (Table 5, *p *< 0.0001).

## Discussion

We took a genomics approach to study metastasis biology and classified patients with breast cancer according to the presence and location of their metastases (that is, MetScore). The resulting analyses showed that the most distinct group with the most distinguishing features were the distant metastases; few differences were seen between primary tumors and regional metastases, as has been shown before [57]. When the set of genes that were correlated with MetScore was determined, many previously known gene sets were identified including proliferation [58], ER status [11,39,40], and fibroblast and/or mesenchymal genes [55,59]. Notable distant metastasis features included the low expression of fibroblast genes (and a corresponding paucity of fibroblasts as defined by histological examination) and the high expression of the VEGF profile. The VEGF profile represents a *in vivo *defined gene expression program that includes a combination of cell-intrinsic and cell-extrinsic factors. The cell-extrinsic factors have known roles as inducers of endothelial cell growth (*VEGF *and *ANGPTL4*), inducers of lymphatic vessel growth (*ADM*) [60], and smooth muscle cell dynamics (*GAL*); thus, the expression of this gene set would appear to increase the likelihood of tumor survival by causing *de novo *vessel formation and providing a dual conduit for metastatic spread. The cell-intrinsic factors include the high expression of *SLC16A3*, whose function is to efflux the lactic acid out of the cell that occurs during high glycolytic activity, and the expression of *NDRG1*, which is a known hypoxia-inducible gene [61,62]. In addition, the tumors that highly express the VEGF profile also highly express glycolytic enzymes. In total, our data suggests poor-outcome distant metastasis samples have the intrinsic ability to promote vessel formation, the intrinsic ability to live under anaerobic conditions, and have lost dependence upon fibroblasts.

Many genomic profiles for breast tumor metastasis biology have been identified, and we therefore compared them with each other and determined that significant correlations exist. In particular, all metastasis profiles tested correlated with 'intrinsic subtype'. For example, the Basal-like subtype showed significant correlation with the 11-gene stem cell profile, the lung and the bone metastasis profiles (consistent with these observations, one of the MetScore 3 Basal-like patients had distant metastases present in the bone, lung and liver). The Basal-like subtype also showed high expression of *Snail*, and Basal-like tumors have been shown to have other features of epithelial-mesenchymal transition [63] including vimentin expression [64].

## Conclusion

The VEGF profile showed very significant prognostic value when using primary tumors, even when tested in models that contained many other expression predictors and clinical variables. We also believe it possible that the VEGF profile may have predictive value for angiogenesis inhibitors because it contains *VEGF *and *ANGPTL4*, which are inducers of angiogenesis. How, or if, the VEGF profile is correlated with response to angiogenesis inhibitors remains to be determined; however, our profile does suggests that effective anti-angiogenesis therapies for patients who express this profile may need to extend beyond VEGF to include the simultaneous targeting of ANGPTL4 and/or ADM.

## Abbreviations

CV: Cross-Validation; DIG: Digoxigenin; DWD: Distant Weight Discrimination; ER: Estrogen Receptor; IHC: Immunohistochemistry; ISH: *In-Situ *Hybridization; MetScore: Metastasis Scoring System; NKI: Netherlands Cancer Institute; RFS: Relapse-Free Survival; SAM: Significance Analysis of Microarrays; UNC: University of North Carolina; VEGF: Vascular Endothelial Growth Factor.

## Competing interests

CMP and ZH have filed a patent application for the use of the VEGF profile for breast cancer prognosis.

## Authors' contributions

ZH, CF, XH, CL, DSO, SS, RW, FI and CMP made substantial contributions to the concepts, acquisition and analysis of the data. ZH, DSO, MGE, LAC, OIO, MVDR and CMP contributed significantly to the drafting of the manuscript and its intellectual content. All authors have read and approved the final manuscript.

## Pre-publication history

The pre-publication history for this paper can be accessed here:



## Supplementary Material

Additional file 1 **Supplementary table. Summary of GEO submission of 202 microarrays used in this paper and clinical data of the patients in this study.** Click here for file

Additional file 2 **Figure S1.** The complete cluster diagram of all 146 patients using the 1195 gene. MetScore-associated gene list. Click here for file

Additional file 3 **Figure S2.** Univariate Kaplan-Meier survival plots for patients stratified using the VEGF profile based upon rank order expression on the A-B) UNC training data set, C-D) NKI test data set, E-F) Bhattacharjee et al [29] lung carcinoma data set, and G-H) Nutt et al [30] glioblastoma data set. Click here for file
