# Supplementary figures and images for: A compact VEGF signature associated with distant metastases and poor outcomes
Source: BMC Med. 2009 Mar 16;7:9. doi: 10.1186/1741-7015-7-9 (PMC2671523; doi:10.1186/1741-7015-7-9)

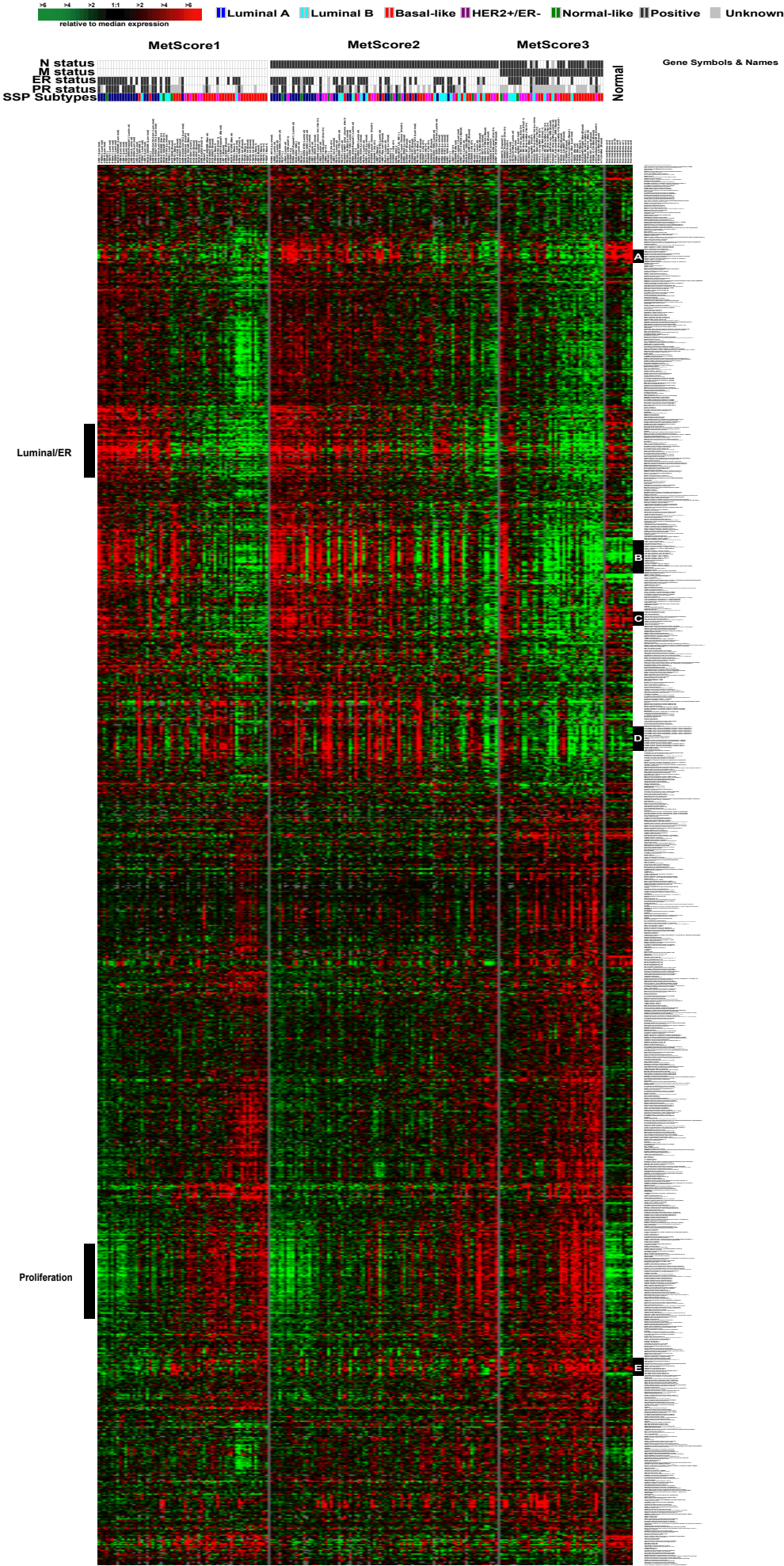

Supplement: Additional file 2 — Figure S1. The complete cluster diagram of all 146 patients using the 1195 gene. MetScore-associated gene list. [file 1741-7015-7-9-S2.pdf]

UNC Training Dataset (N=136)

NKI Test Dataset (N=295)

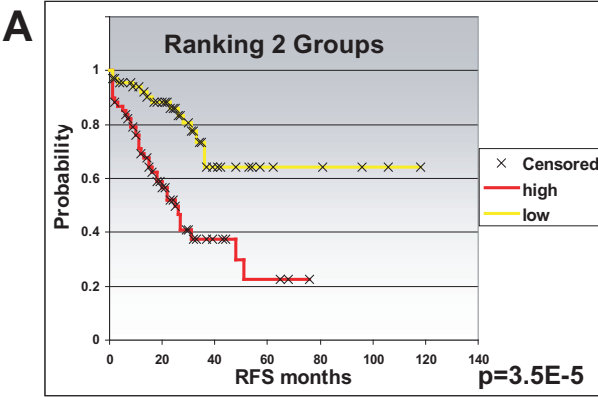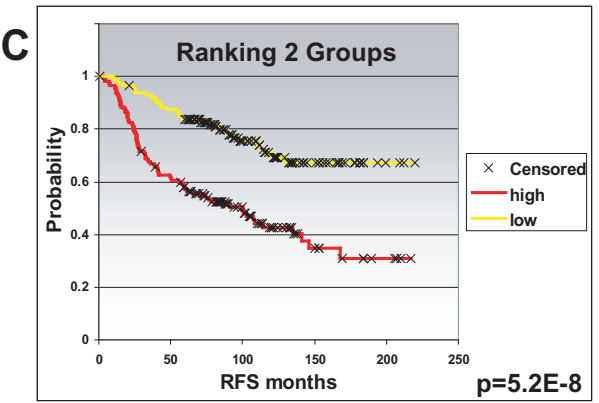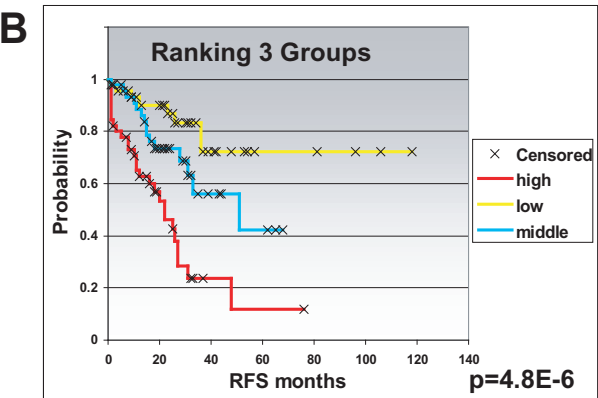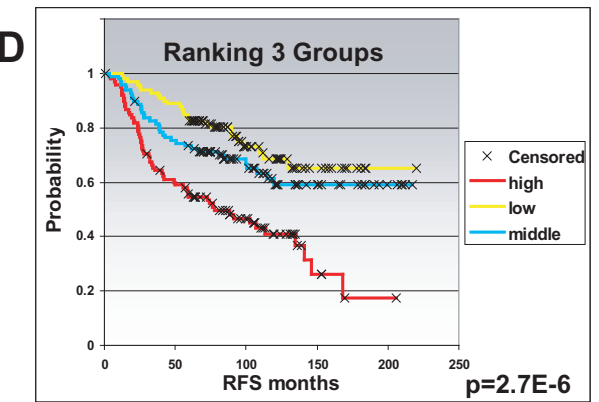

Bhattacharjee et al Lung Cancer (N=111)

Nutt et al. Glioma (N=50)

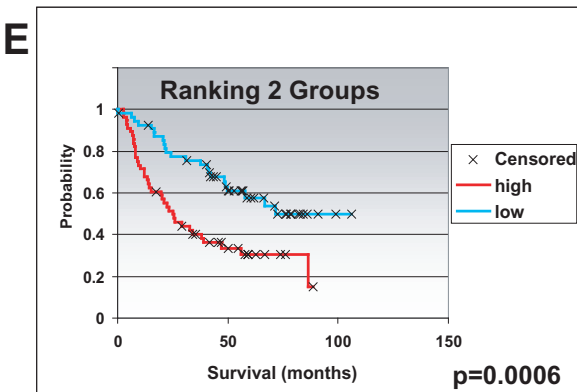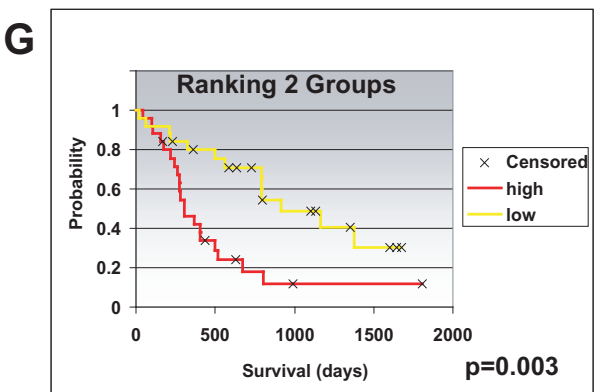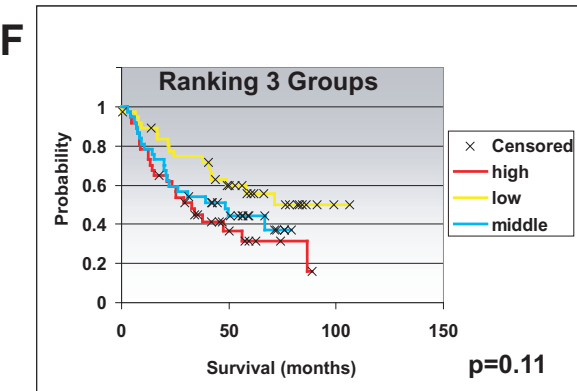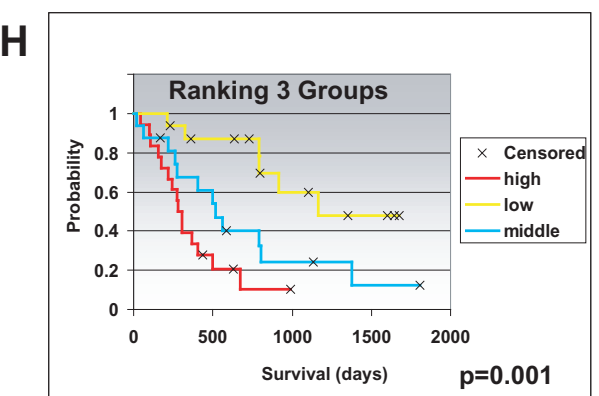

Supplement: Additional file 3 — Figure S2. Univariate Kaplan-Meier survival plots for patients stratified using the VEGF profile based upon rank order expression on the A-B) UNC training data set, C-D) NKI test data set, E-F) Bhattacharjee et al [29] lung carcinoma data set, and G-H) Nutt et al [30] glioblastoma data set. [file 1741-7015-7-9-S3.pdf]
